# From Waste to Value: Optimization of Ultrasound-Assisted Extraction of Anthocyanins and Flavonols from *Pistacia lentiscus* L. Oilcakes

**DOI:** 10.3390/molecules30020237

**Published:** 2025-01-09

**Authors:** Lucrezia Muti, Luana Beatriz dos Santos Nascimento, Giulia Goracci, Cassandra Detti, Cecilia Brunetti, Anna Rita Bilia, Francesco Ferrini, Antonella Gori

**Affiliations:** 1 Department of Agriculture, Food, Environment and Forestry (DAGRI), University of Florence, Sesto Fiorentino, 50019 Florence, Italy; lucrezia.muti@unifi.it (L.M.); cassandra.detti@unifi.it (C.D.); cecilia.brunetti@ipsp.cnr.it (C.B.); francesco.ferrini@unifi.it (F.F.); antonella.gori@unifi.it (A.G.); 2 Department of Chemistry, University of Florence, Via U. Schiff 6, Sesto Fiorentino, 50019 Florence, Italy; giulia.goracci@unifi.it (G.G.); ar.bilia@unifi.it (A.R.B.); 3 Institute for Sustainable Plant Protection (IPSP), National Research Council of Italy, Sesto Fiorentino, 50019 Florence, Italy

**Keywords:** lentisk, oilcake, green extraction, design of experiment, waste recovery

## Abstract

*Pistacia lentiscus* L., commonly known as the mastic tree or lentisk, is a woody Mediterranean plant revered for its ecological relevance as well as for its extensive ethnobotanical heritage. Historically, the fruits and the resin of *P. lentiscus* have been widely utilized in traditional medicine, underscoring its important role in local healing practices. Given these properties, this study explored an innovative approach to efficiently extract anthocyanins and flavonols from *P. lentiscus* oilcakes utilizing ultrasound-assisted extraction (UAE) as an alternative to conventional solvent extraction. Liquid chromatography–mass spectrometry (LC-MS) and high-performance liquid chromatography with diode-array detection (HPLC-DAD) were used to identify and quantify the anthocyanins and flavonols, revealing the successful extraction of eight distinct anthocyanins and twenty flavonols. A Fractional Factorial Design (FFD) followed by a Box–Behnken design (BBD) were applied to optimize the yield of anthocyanins and flavonols. The optimal extraction conditions found were to be an extraction time of 15 min with 70% ethanol as the solvent and a liquid-to-solid ratio of 0.012 L g^−1^, which resulted in a maximum extraction yield of 19.78 mg g^−1^ dry extract for the Total Flavonol Content and over 25.4 mg g^−1^ dry extract for the Total Flavonol and Anthocyanin Content. By elucidating the optimal conditions for extracting anthocyanins and flavonol glycosides, this study opens promising avenues for utilizing *P. lentiscus* oilcake by-products, supporting sustainable practices, and advancing the valorization of Mediterranean bio-resources for health-promoting applications.

## 1. Introduction

Plant preparations have been used in folk medicine and ethnobotany since ancient times. Nowadays, there is growing attention towards revisiting plant materials for drug discovery, with scientific research increasingly validating their role in treating different diseases [1,2]. Among the Mediterranean species, *Pistacia lentiscus* L. (PL, Anacardiaceae) is an evergreen woody species with a long ethnobotanical tradition [3]. Since ancient times, its aerial parts have been utilized to prevent and treat various ailments, including hypertension, asthma, allergies, skin problems, respiratory difficulties, and kidney problems [4].

The research has extensively focused on the chemical properties and biological activities of aerial parts of this plant. The leaves are known for their strong antioxidant properties, mainly due to the presence of gallic acid and catechin derivatives, which contribute to their significant free radical scavenging and anti-inflammatory activities [5]. Mastic preparations, on the other hand, are valuable in treating digestive problems, skin diseases, and wounds [6]. Furthermore, the European Medicines Agency (EMA) has officially recognized PL resin as a traditional herbal medicine, specifically endorsing its use in treating mild dyspeptic disorders [7].

While the leaves and mastic preparations of PL are well studied, the fruits have been relatively understudied despite their widespread use in folk medicine and culinary traditions, particularly in Tunisia and Sardinia [8,9]. Historically, the drupes have been processed into edible oils, valued for their nutritional quality and ability to preserve and enrich the taste of various dishes [10]. PL oil has a high triacylglycerol content, mainly oleic, palmitic, and linoleic acids, and also contains aliphatic alcohols, including dotriacontan-1-ol and triacontan-1-ol [11]. In addition, PL fruits and their oil are rich in polyphenols, tocopherols, carotenoids, and essential oils [8,12], as well as terpenes and saponins [13]. In terms of polyphenols, flavonoids (mainly catechins, quercetin, and galloyl derivatives) and anthocyanins (primarily delphinidin and cyanidin derivatives) are the primary compounds described in fruits. These molecules are renowned for their antioxidant properties and play significant roles in human health, including anti-inflammatory and anti-cancer properties [14,15,16]. In addition, anthocyanins and flavonoids are used as natural colorants and preservatives in the food industry thanks to their stability and health benefits [17]. In cosmetics, these compounds are incorporated into many products for their protective effects against UV radiation and oxidative stress, improving skin health and appearance [18]. Moreover, in the pharmaceutical sector, their bioactive properties are exploited in formulations aimed at preventing chronic diseases and promoting overall well-being [17]. Thus, the ability of these compounds to modulate various biological pathways makes them highly valuable ingredients in functional foods, nutraceuticals, and therapeutic supplements.

Although PL oil has known applications as a nutritional and traditional remedy [19,20], it accounts for only 30% of the fruit weight, leaving almost 70% as waste after oil extraction. This waste, hereafter referred to as PL oilcakes, consists mainly of fruit skins, seeds, and pulp, and is typically produced after the traditional cold pressing of PL oil from fresh fruits harvested during the winter season [21,22].

In recent years, there has been increasing interest in the reutilization of agri-food waste, including oilcakes derived from lentisk oil production. Polyphenols extracted from lentisk oilcakes have been tested for their potential antidiabetic activity [23], underscoring the presence of valuable bioactive compounds. Additionally, lentisk oilcake extracts have been applied as natural emulsifiers in beta-carotene-enriched nanoemulsions, demonstrating their versatile functionality [24]. These studies highlight the potential of lentisk oilcakes as a rich source of bioactive molecules, including flavonoids and anthocyanins, emphasizing the need to optimize extraction processes to achieve higher yields.

The extraction of phenolic compounds from plant waste materials presents challenges due to the complex structure of plant matrices and the biochemical instability of these compounds. Among the various extraction methods, ultrasound-assisted extraction (UAE) has demonstrated significant efficacy in enhancing both the yield and purity of flavonoids and anthocyanins from plant sources. UAE leads to higher yields of phenolic compounds in a shorter time compared to traditional extraction methods [25]. As a result, green extraction techniques can be applied to extract bioactive compounds from plant industrial by-products, giving a second life to the biomass while reducing waste. Therefore, the UAE method can be considered a waste-to-wealth strategy to transform natural waste materials into valuable bioactive compounds that can be applied in the health, food, and agronomic industries.

In this context, our study aimed to valorize *Pistacia lentiscus* L. oilcakes by optimizing the extraction conditions using an eco-friendly UAE technique to obtain a flavonoid-enriched extract that can be applied in the cosmetic, phytochemical, and food industries. The effect of UAE conditions on the flavonol and anthocyanin contents was investigated using Response Surface Methodology (RSM), a powerful tool for developing mathematical models to optimize multiple variables and maximize extraction efficiency. This approach supports sustainable practices by minimizing waste and helps in identifying new bioactive resources.

## 2. Results and Discussion

### 2.1. Chemical Characterization of Pistacia lentiscus Oilcake Extracts

**Figure 1:**
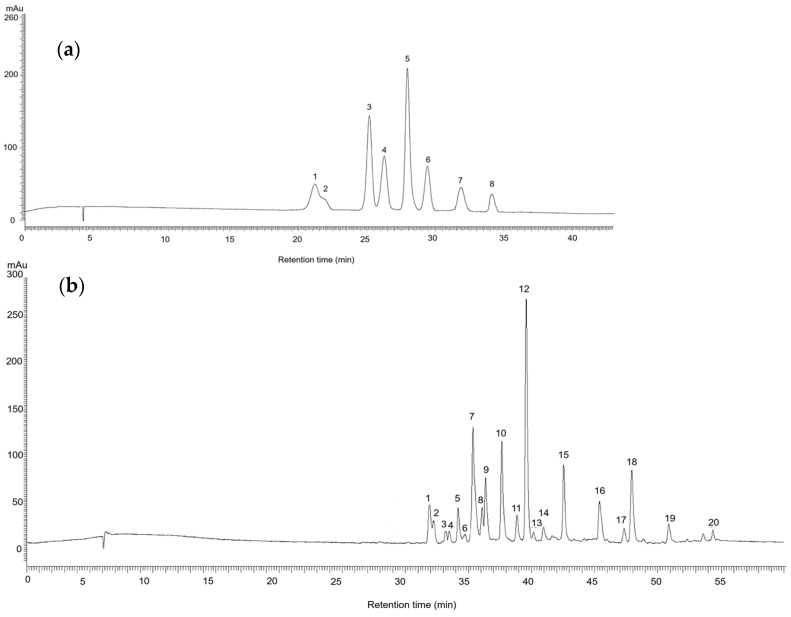
HPLC-DAD chromatograms of the richest PL oilcake extracts. (**a**) Anthocyanin chromatogram from the AF detected at λ 520 nm, and (**b**) flavonol chromatogram from the EF detected at λ 350 nm. Individual compound identifications are listed in Table 1.

The HPLC-DAD and LC-MS analyses allowed for the identification of twenty-eight phenolic compounds in the PL oilcake extract fractions, including eight anthocyanins from the AF (delphinidin and cyanin derivatives) and twenty flavonols from the EF (mainly myricetin, quercetin, and kaempferol derivatives). The identified compounds are listed in Table 1, which correspond to the peaks shown in the HPLC-DAD representative chromatogram in Figure 1.

#### 2.1.1. Identified Anthocyanins from Aqueous Fraction

As reported in Table 1, the anthocyanin fraction of the PL oilcake extract was composed of eight glycosylated anthocyanins belonging to two major classes: cyanidin and delphinidin derivatives. These included two di-hexoses and six mono-glycosides, with the latter encompassing two pentose derivatives. In PL fruits, as in other plant sources, anthocyanins not only contribute to the visual appeal but also possess significant health-promoting properties due to their potent antioxidant activities [26]. Indeed, it has been demonstrated that glycosylated anthocyanins can have a strong inhibition effect on colon-related cancer, reducing glucose uptake in tumor cells and leading to apoptosis [27]. Furthermore, since anthocyanins are polar pigments, ethanolic extractions have higher yields compared to methanolic ones. Moreover, mineral acids are often added to the extraction solvent to stabilize the flavylium cation and to regulate their color, preventing their degradation and making the extraction more efficient [28].

Peaks 1 and 2 shared the same aglycon [M-H]^+^ at *m*/*z* 303, corresponding to the anthocyanidin delphinidin, and two fragment ions [M-H]^+^ at *m*/*z* 627 and [M-H]^+^ at *m*/*z* 465, indicating two-hexose losses. For this reason, they were tentatively identified as two di-glycosylated isomers of delphinidin. Peaks 3 and 4 were identified as delphinidin 3-*O*-galactoside and delphinidin 3-*O*-glucoside, exhibiting the same precursor ion [M-H]^+^ as peaks 1 and 2 at *m*/*z* 303 and a corresponding fragment ion [M-H]^+^ at *m*/*z* 465 (−162, hexose loss). The identities were further confirmed through their different retention times and the injection of the external standards.

Peaks 5 and 7 were identified as two cyanidin derivatives, cyanidin-3-*O*-galactoside and cyanidin-3-*O*-glycoside, respectively. Both exhibited the same aglycon [M-H]^+^ at *m*/*z* 287 corresponding to cyanidin aglycone and the same parent ion [M-H]^+^ at *m*/*z* 449 with a neutral loss of one hexose moiety (*m*/*z* 162). The characteristic neutral loss of a hexose moiety (*m*/*z* 162) from the parent ion [M-H]^+^ (*m*/*z* 449) is consistent with the known fragmentation patterns for cyanidin derivatives [29]. Cyanidin-3-*O*-galactoside was the most abundant compound in the AF. The presence of cyanidin-3-*O*-glucoside was previously found in *Pistacia vera* nuts. Moreover, the identity of peak 7 was further confirmed by the injection of the corresponding external standard.

Peak 6 displayed the aglycon [M-H]^+^ at *m*/*z* 303 (delphinidin aglycone) along with a fragment ion [M-H]^+^ at *m*/*z* 435, indicating a neutral loss of a pentose moiety (*m*/*z* 132). For this reason, it was tentatively classified as delphinidin pentoside.

Peak 8, sharing the same aglycon as peaks 5 and 7 ([M-H]^+^ at *m*/*z* 287), with a parent ion [M-H]^+^ at *m*/*z* 419 and a neutral loss of a pentose moiety (*m*/*z* 132), was identified as cyanidin pentoside, based on other studies on PL [30].

#### 2.1.2. Identified Flavonols from Ethylacetate Fraction

Twenty glycosylated flavonols were identified from the EF: seven myricetin derivatives, eight quercetin derivatives, and four kaempferol derivatives, as reported in Table 1. The flavonols in the EF were identified based on the mass fragmentation pattern of their corresponding aglycon units: myricetin ([M-H]^−^ at *m*/*z* 317), quercetin ([M-H]^−^ at *m*/*z* 301), and kaempferol ([M-H]^−^ at *m*/*z* 285). The sugar loss was characterized based on neutral losses of 132 (pentose moiety), 146 (rhamnose moiety), 162 (hexose moiety), and 308 (rhamnoglucoside moiety). The identification was confirmed by the injection of external standards, when available, along with the fragmentation patterns in the scientific literature [31].

Peak 1 was identified as myricetin rutinoside, whose mass spectra showed a precursor ion [M-H]^−^ at *m*/*z* 625 with an MS^2^ fragmentation ion [M-H]^−^ at *m*/*z* 479, indicating a loss of a rhamnose moiety (*m*/*z* 146) and a second loss of a glucose moiety (*m*/*z* 162), resulting in the fragment [M-H]^−^ of myricetin aglycon at *m*/*z* 317. The identification of myricetin rutinoside, based on the loss of rhamnose and glucose moieties, aligns with the fragmentation patterns reported in previous studies, such as the one by Bishbishy et al. [32].

Peak 2 was identified as myricetin glucuronide, presenting a precursor ion [M-H]^−^ at *m*/*z* 493 and a fragment ion [M-H]^−^ at *m*/*z* 317, the latter corresponding to the aglycone of myricetin, with a neutral loss of glucuronic acid (*m*/*z* 176).

Peaks 3 and 4 similarly showed a fragment ion [M-H]^−^ at *m*/*z* 317, indicating a myricetin aglycone associated with a precursor ion [M-H]^−^ at *m*/*z* 479. The difference between the two fragments indicated a neutral loss of *m*/*z* 162, corresponding to a hexose moiety. For this reason, their identity was indicated as myricetin-hexose isomers 1 and 2. Their putative identification was supported by their UV spectra, with both possessing a λ_max_ of absorbance at 360 and 270 nm, with a shoulder at 300 nm.

Peaks 5 and 6 showed a precursor ion [M-H]^-^ at *m*/*z* 463, associated with a fragment ion [M-H]^−^ of the quercetin aglycone at *m*/*z* 301. Furthermore, the UV spectra presented a λ_max_ at 350 nm and 278 nm. For this reason, they were tentatively identified as quercetin-3-*O*-glucoside isomers 1 and 2. Similarly, the identification of quercetin-3-*O*-glucoside isomers and rutin, a common flavonoid in many plant species, corroborates the findings of previous research on *Pistacia lentiscus* L. [33] and *Pistacia vera* L. [34].

Peak 7 was identified as a myricetin pentose, presenting a precursor ion [M-H]^−^ at *m*/*z* 449 and a fragment ion [M-H]^−^ of a myricetin aglycone at *m*/*z* 317. Other studies in PL leaves already described the presence of pentosyl derivatives of myricetin [35], particularly myricetin-3-*O*-rhamnoside.

Peak 8 exhibited a precursor ion [M-H]- at *m*/*z* 447 and a fragment ion [M-H]^−^ at *m*/*z* 301, indicating a pentose neutral loss. For this reason, it was identified as quercitrin (quercetin-3-rhamnoside), and the same molecule was detected in a PL fruit extract by [33]. Peak 9 was assigned to rutin, also known as quercetin-3-*O*-rutinoside, which exhibits a specific fragmentation pattern involving the cleavage of the glycosidic bond that binds the rutinose sugar (composed of glucose and rhamnose) to the quercetin aglycone, which results in the loss of the rutinose moiety as a neutral molecule from the precursor ion [M-H]^−^ at *m*/*z* 609 and the detection of the resulting quercetin aglycone at *m*/*z* 301. In addition, the fragmentation pattern of the rutinose disaccharide produced an additional fragment ion [M-H]^−^ at *m*/*z* 463, indicating a loss of a rhamnose moiety from the precursor ion. Other research detected rutin in PL fruits and leaves [32].

Peak 10 was tentatively identified as myricitrin (myricetin 3-*O*-rhamnoside) due to the presence of a characteristic fragment ion at *m*/*z* 317 and a precursor ion at *m*/*z* 463, indicating a neutral loss of a rhamnose (*m*/*z* 146). Peak 11 was assigned as quercetin-pentoside, thanks to the precursor ion [M-H]^−^ at *m*/*z* 433 and a fragment ion [M-H]^−^ at *m*/*z* 301, suggesting a neutral loss of pentose moiety (*m*/*z* 132). Furthermore, the signal at *m*/*z* 867 (double + 1) confirmed the putative identification [35]. Based on the fragmentation pattern, peak 12 was tentatively identified as kaempferol derivative, showing the fragment ion [M-H]^−^ at *m/z* 285.

The identity of peak 13 instead was tentatively assigned with myricetin-derivative, presenting a precursor ion [M-H]^−^ at *m*/*z* 469 and a fragment ion [M-H]^−^ at *m*/*z* 317, with a neutral loss of *m*/*z* 152. Peaks 14 and 16 were putatively identified as kaempferol derivatives, sharing the fragment ion [M-H]^−^ at *m*/*z* 285 corresponding to kaempferol aglycone, while peak 15 has been putatively identified as a flavonoid derivative with the main ion [M-H]^−^ at *m*/*z* 269. Peak 18 was identified as quercetin aglycone, with a [M-H]^−^ signal at *m*/*z* 301. Lastly, peaks 17, 19, and 20 were assigned as kaempferol derivatives, as they showed the kaempferol aglycone [M-H]^−^ at *m*/*z* 285 and similar UV–Vis spectra.

Our findings are consistent with a previous study on PL conducted by Remila et al. [36] that described six glycosylated flavonols in a PL leaf extract: myricetin-rutinoside, myricetin-glucoside, quercetin-rutinoside, myricetin rhamnoside, quercetin, glucoside, and quercetin-rhamnoside. Glycosylation often reduces flavonols’ immediate bioactivity in vitro and it can enhance flavonoid stability, solubility, and metabolism, potentially improving their health benefits in vivo, particularly through interactions with the gut microbiome and their extended bioavailability [37].

In conclusion, the comprehensive LC-MS analysis of the EF obtained from a PL oilcake revealed a diverse profile of twenty glycosylated flavonols, including derivatives of myricetin, quercetin, and kaempferol, confirming both the complexity and variety of flavonol glycosides. The observed glycosylation patterns, as well as their fragmentation profiles, align with those of previous studies [30,31,38], suggesting a conserved structural diversity among flavonoids in *P. lentiscus* L. Moreover, the glycosylation process may play a crucial role in enhancing the stability, bioavailability, and health-promoting potential of flavonoids, making them more effective for human dietary consumption and supplementation [39]. In fact, while flavonoid aglycones exhibit a higher absorption rate and may demonstrate stronger biological activity, glycosylated flavonoids offer enhanced solubility and stability in water. In fact, while glycosylation can mask hydroxyl groups—reducing the intrinsic radical-scavenging capacity—it plays an important role in enhancing bioavailability and absorption in vivo due to the presence of sugar moieties, which improve their dispersion in aqueous environments. This process is crucial because flavonoids typically have poor water solubility, which limits their bioavailability and effectiveness as nutraceuticals and stability as pharmaceuticals [40]. Furthermore, the enhancement of bioavailability is achieved by enabling gut microbiota to metabolize glycosides into their more active aglycone forms, which exhibit potent antioxidant properties [41,42].

### 2.2. Screening and Optimization Designs

#### 2.2.1. Screening Design

All the extracts obtained from the different trials of the screening and optimization design showed the same qualitative anthocyanin (AF) and flavonol (EF) profiles, while significant quantitative differences were observed between them (see Tables S1 and S2 in Supplementary Materials).

Numerous factors can influence the extraction of secondary metabolites from plant materials, including the solvent type, extraction time, temperature, ultrasound power, liquid-to-solid ratio, and specific method used. Additionally, the characteristics of the plant material play a crucial role, including variables such as the geographical area, environmental conditions, period of harvesting, and seasonality, which significantly affect the polyphenol metabolic profile [43]. Emerging research has reported high recovery rates of polyphenols from biomass residues such as almond hulls, olive leaves, and orange and pomegranate peels [44,45,46]. UAE is one of the fastest, most cost-effective, and most lab-friendly techniques compared to conventional extraction methods (e.g., maceration, Soxhlet, and infusion) [47]. This approach minimizes the environmental impact and enhances resource efficiency, as outlined in key green chemistry principles. UAE also enables the use of eco-friendly solvents like ethanol–water mixtures, which are widely applied as solvents for polyphenol extraction from plant matrices [48,49]. However, it is important to mention that some trade-offs exist when scaling UAE for industrial applications. While UAE is resource-efficient, its scalability might face challenges such as higher equipment costs for large-scale ultrasonic transducers and the need for uniform energy distribution in larger volumes. Additionally, operational parameters, including solvent recovery and maintenance, must be optimized to maintain efficiency at industrial scales. Among the many variables in the UAE method, the temperature, ethanol concentration, time, and liquid-to-solid ratio are the ones with the most influence on polyphenol extraction.

In our study, a subset of the full factorial design (2^4−1^) was strategically chosen to investigate the main effects and specific interactions among variables, enabling the identification of the ones with significant contributions to the extraction process. The independent variables of the UAE screening design were the temperature (25–50 °C), time (10–30 min), solvent (10–50% *v*/*v*), and liquid-to-solid ratio (0.01–0.02 L g^−1^). The matrix with coded and uncoded variables, and the calculated coefficients for the TFC, TAC, and TFAC obtained from the screening step are reported in Table 2.

The analysis of the calculated coefficients showed a negative and statistically significant effect of the time (x_2_) on the TFC and TFAC. A decrease in extraction time (x_2_) increased the TFC and TFAC. In addition, the b_2_ coefficient’s absolute value for the TFC was higher than that for TAC, indicating a greater magnitude of effect on the TFC in comparison to the TAC. Using a short extraction time is cost-effective and beneficial for maintaining the stability of thermolabile compounds such as polyphenols and anthocyanins. Shorter extraction times reduce their degradation caused by prolonged exposure to ultrasonic cavitation, heat buildup, or oxidative stress [50]. Furthermore, reducing the extraction time minimizes the use of resources such as electricity or cooling systems, further lowering the operational costs.

The coefficient b_3_, corresponding to the extraction solvent (% *v*/*v* EtOH, x_3_), had a statistically significant effect on the TAC. Its positive value indicates that, as the concentration of ethanol rose, the total anthocyanin yield increased significantly. The efficiency of ethanol in extracting anthocyanins is linked to its ability to disrupt cell membranes and solubilize these compounds, which is depended on its concentration.

The b_4_ coefficient, corresponding to the liquid-to-solid ratio (LSR, x_4_), also had a significant effect on the TFC and TFAC. For both responses, the higher the LSR, the lower the TFC and TFAC. Increasing the volume of extraction solvent relative to the plant material generally leads to an enhanced yield of flavonoids, which is attributable to the increased solubility of these compounds. Similar results were found by Hao et al. in UAE of flavonoids from *Lactuca indica* L. [51]. However, an excessive volume of solvent may extend the diffusion distance of the compounds between the tissues and the solvent, hindering their complete solubilization and, consequently, extraction efficiency. The inverse relationship between the LSR, TFC, and TFAC in our study aligns with the findings of Elboughdiri et al. [52] who reported that a higher solvent-to-solid material ratio might reduce the concentration of the target compounds, thus lowering the overall extraction efficiency.

The temperature (x_1_), associated with the b_1_ coefficient, did not show a statistically significant effect on the outputs. Therefore, the temperature was excluded as a variable factor in the optimization step, and was kept constant at room temperature. Maintaining room temperature during the extraction process is essential, as high temperatures can negatively affect both the chemical composition of the extract and the overall costs of large-scale operations. Additionally, heating large volumes of solvents or plant materials over extended periods requires substantial energy, driving up operational costs and making the process less efficient and economically viable for large-scale operations. Studies, such as those conducted by Cacace et al. [53] and Modesto et al. [54], have shown that higher temperatures compromise the chemical stability of sensitive compounds such as anthocyanins, leading to their accelerated degradation and to a loss of nutritional value, in addition to health benefits.

Overall, utilizing the screening design as a first step in the extraction process reduces the experiment effort by narrowing down the number of critical variables and reducing the complexity of the optimization design. Furthermore, this is crucial in green extractions where minimizing resource use (e.g., energy, solvents, and time) is a priority [55].

#### 2.2.2. Optimization Design and Response Surface Methodology

Based on the results from the screening step, a Box–Behnken design was used to conduct the optimization of the extraction conditions. Table 3 displays the Box–Behnken design matrix, providing both coded and uncoded values for the independent variables and the outputs. The independent variables in the UAE optimization were the time (5–15 min), solvent (30–70% *v*/*v*), and liquid-to-solid ratio (0.008–0.012 L g^−1^). The content of the individual identified compounds in the AF and EF in the optimization design are reported in the Supplementary Materials (Tables S1 and S2).

The solvent composition had a significant effect on all three outputs (*p* ≤ 0.05), as shown by the calculated coefficients in Table 3. Among the tested conditions, run 4 (15 347 min, 70% EtOH, and 0.01 g L^−1^) yielded the highest TFC and TAC values, alongside the highest TFAC, with cyanidin-3-*O*-galactoside and kaempferol derivative being the most abundant compounds in the AF and EF, respectively. Similarly, run 3 (5 min, 70% EtOH, and 0.01 g L^−1^) also resulted in high yields, with the only variation between these runs being the extraction time (x_1_), whose associated coefficient (b1) was not statistically significant. Notably, run 10 achieved high TAC values (5 mg g^−1^), attributed to the maximum ethanol concentration (70%). A comparable result was seen in run 8, where the high EtOH percentage and increased LSR resulted in a high TAC (5.2 mg g^−1^). However, despite the high TAC in run 8, the TFC was lower compared to runs 3, 4, and 10, leading to a lower overall TFAC.

Runs 5 and 6, for which the EtOH percentage and LSR were kept at 50% *v*/*v* and 0.008 L g^−1^, respectively, revealed that switching from the shortest extraction time (5 min) to the longest time (15 min) resulted in similar values for the TFC, TAC, and TFAC. This observation suggests that extraction time had a minimal impact on the outputs under these conditions, further supporting the notion that the other factors, like the solvent concentration and LSR, play a more significant role in determining the extraction efficiency.

Moreover, when the ethanol percentage was reduced to a minimum (30% *v*/*v*), as in runs 1, 2, 9, and 11, the TFC, TAC, and TFAC were consistently low. In contrast, using 50% ethanol, coupled with a higher solvent ratio (LSR = 0.012 L g^−1^) and an extended extraction time (15 min), as seen in runs 7 and 8, led to elevated values for the TFC, TAC, and TFAC. This emphasizes the role of the LSR over longer extraction times in enhancing the TFAC, further supported by the statistically significant coefficient *b13* (see Supplementary Materials, Table S3).

Additionally, the ethanol concentration was a key determinant in the extraction efficiency from the PL oilcake, with the optimal results observed in runs where 70% *v*/*v* EtOH was utilized. Lastly, runs 1 and 2, which only differed in extraction time (5 vs. 15 min), confirmed that extending the process duration was not sufficient to significantly boost the TFC, TAC, or TFAC when working with lower ethanol percentages.

Beyond this point, a prolonged extraction time may not significantly enhance the yield, possibly due to the saturation of the solvent or the exhaustion of easily extractable compounds. In addition, a shorter extraction time could be more cost-effective and energy-efficient without compromising the yield of valuable bioactive compounds. The time-saving extraction can be beneficial for scaling up the process in industrial applications, where minimizing operational time while maximizing extraction efficiency is a key factor in reducing costs and environmental impacts.

The statistical data analysis yielded valuable insights into the relationship between the key independent variables—time, solvent, and liquid-to-solid ratio—and the outputs, as reported in Table 4. Regarding the TFC, the model demonstrated good explanatory power, with a coefficient of determination (R^2^) of 0.99, indicating that the independent variables effectively elucidated the variance in the response (TFC values). The adjusted R^2^ (0.96) further confirmed the model’s goodness of fit. The highly significant F-value (43.36) and low *p*-value (0.001) underscore the reliability of the model, and the lack of fit (LOF) analysis indicated a reasonable fit with an F-value of 2.55 and a *p*-value of 0.294. Similarly, the TAC model exhibited a substantial explanatory capability, with an R^2^ of 0.92, an adjusted R^2^ of 0.78, and a significant F-value (6.61; *p* = 0.026). No lack of fit was observed (*p* = 0.214), showing a satisfactory fit of the model. Lastly, the model for the TFAC mirrored the TFC model in terms of a high R^2^ (0.99) and adjusted R^2^ (0.98), significant F-value (64.77), and low *p*-value (0.001). The corresponding LOF analysis indicated a well-fitting model with an F-value of 4.37 and a *p*-value of 0.192. These results collectively reveal the robustness of the models for predicting the flavonol and anthocyanin content in PL oilcake extracts, therefore providing valuable tools for optimizing the extraction conditions while highlighting the significance of the independent variables in shaping these outcomes.

**Figure 2:**
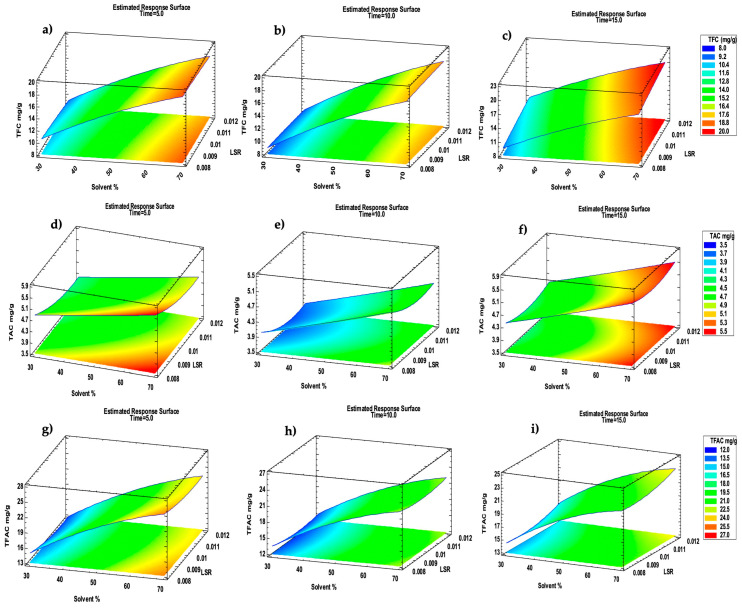
RSM plots of Box–Behnken design displaying how solvent % (x_2_) and liquid-to-solid ratio (x_3_) can influence TFC (**a**–**c**), TAC (**d**,**e**), and TFAC (**g**–**i**). (**a**–**c**) TFC at 5 min (**a**), 10 min (**b**), and 15 min (**c**); (**d**–**f**): TAC at 5 min (**d**), 10 min (**e**), and 15 min (**f**); (**g**–**i**) TFC at 5 min (**g**), 10 min (**h**), and 15 min (**i**). Results are expressed as mg g^−1^ of lentisk oilcake extract for TFC (Total Flavonol Content), TAC (Total Anthocyanin Content), and TFAC (Total Flavonol and Anthocyanin Content. Red region shows working region with maximum yield.

The 3-D Response Surface Methodology (RSM) graphs shown in Figure 2 were built according to the TFC, TAC, and TFAC polynomial models and they illustrate the variation in the TFC, TAC, and TFAC with the ethanol concentration (x_2_) and LSR (x_3_) while keeping the time (x_1_) at a fixed duration.

The optimal UAE conditions (Figure 2c,i) suggested by the model were an extraction time (x_1_) of 15 min, using 70% EtOH (x_2_) and a liquid-to-solid ratio (x_3_) of 0.012 L g^−1^, resulting in the maximum TFC and TFAC at 19.78 mg g^−1^ and 25.4 mg g^−1^ of dry extracts, respectively. The corresponding extraction yield is reported in the Supplementary Material in Table S4.

When considering the two classes of compounds separately, the extraction conditions were slightly different. In fact, regarding the TAC, the optimal extraction conditions were 15 min, 65% EtOH, and 0.01 L g^−1^ for the liquid-to-solid ratio, with a prediction of 5.4 mg g^−1^ for the TAC (Figure 2f). These results indicate that the ethanol concentration was the main factor that affected the outputs (Table 3).

It is noteworthy to mention that similar recent work on PL oilcakes was conducted by Ouatmani et al. [23]. The authors optimized the ultrasound-assisted extraction of phenolic compounds, identifying the optimal conditions as 50% ethanol, 60 °C, and 86 min of sonication, and predicting a Total Phenolic Compound (TPC) of 99.59 mg GAE/g DW. However, while the TPC was the main target, the study did not deeply investigate how different phenolic subclasses (such as anthocyanins and flavonols) responded to the extraction parameters tested. Furthermore, the authors evaluated the total phenolic content by using the Folin–Ciocalteu assay. This method is not specific for phenolic compounds, since the reagent can react with other reducing agents present in plant matrices, such as ascorbic acid and proteins. The lack of specificity can lead to an overestimation of the phenolic content, as the assay cannot differentiate between phenolics and other substances with similar redox potential [56]. In contrast, our LC-MS and HPLC-DAD analyses provided detailed information on specific polyphenols present in PL oilcake extracts, including the concentration and specific structure of the flavonols and anthocyanins, which is crucial for understanding the potential bioactivity and health benefits of this waste, an aspect that is valued and recommended by many industries.

A moderate ethanol–water mixture (e.g., 50–70% *v*/*v* EtOH) often provides an optimal solvent system for flavonoid extraction by balancing solubility and penetration into the plant matrix. Water, as a polar solvent, dissolves hydrophilic compounds, including anthocyanins, while ethanol improves the solubility of flavonols. However, higher ethanol concentrations (e.g., up to 70% *v*/*v*) can decrease the solvent polarity, reducing the solubility of anthocyanins. Therefore, it is important to define a balanced ethanol–water mixture that creates a solvent system that effectively disrupts hydrogen bonding and van der Waals forces between molecules and the fruit matrix, facilitating their release. Our findings align with those of previous studies, such as [30], which also identified 70% ethanol as the most effective concentration for extracting high yields of anthocyanins and flavonoids from PL fruits. While they identified delphinidin 3-*O*-galactoside as the predominant anthocyanin in fruit extracts, our analysis revealed cyanidin-3-*O*-galactoside as the most abundant anthocyanin. These differences may be due to various factors, including geographical and seasonal variations, differences in solvent extraction methods, and the use of different PL raw materials (e.g., fruits instead of oilcakes). Similarly, Chaabani et al. [57] confirmed that a 70% ethanol–water solution is optimal for extracting polyphenols from PL, further supporting our results.

It is important to mention that higher percentages of EtOH (e.g., more than 70% *v*/*v*) can extract other components of PL oilcakes (e.g., saponins), making large-scale extraction processes challenging, as they are natural emulsifiers and surfactants [24].

The RSM study represented in Figure 2 showed that using the minimum time (x_1_, 5 min) with 70% *v*/*v* solvent (x_2_) can yield a good TAC if the LSR (x_3_) is low; when considering the TFC, a good concentration can be reached even with a higher LSR (Figure 2a). When the extraction time (x_1_) is at its maximum, together with the maximum ethanol concentration (x_2_), the TFC and TAC reached the highest values, even with shifting the LSR between the maximum and minimum values.

Extracting flavonoids and anthocyanins from PL oilcakes offers benefits from both industrial and sustainability perspectives, aligning with the principles of the circular economy. As a by-product of the oil extraction process, oilcakes can serve as raw materials for bioactive compound extraction, minimizing waste and supporting circular economy models. This approach is particularly relevant in small Mediterranean regions, where PL fruits are still used as a source of edible oil [58]. Furthermore, using oilcakes for secondary metabolite extraction can reduce the raw material costs for industries producing high-value bioactive compounds, such as flavonols, thus maximizing the economic value of PL fruits by utilizing their by-products. This waste recovery reduces the reliance on fresh plant materials and provides a cost-effective alternative for nutraceutical, cosmetics, and functional food production [59].

## 3. Materials and Methods

### 3.1. Chemicals and Reagents

Formic acid (HCOOH), methanol (Me-OH), ethyl acetate (Et-AcO), milli-Q_H2O_, *n*-hexane, and acetonitrile (ACN) were all analytical grade and were obtained from Sigma Aldrich^®^–Merck^®^KGaA (Darmstadt, Germany). Myricetin, rutin, quercetin-3,4-di*O*-glucoside, quercetin-3-*O*-glucopyranoside, kaempferol-3-*O*-glucoside, kaempferol-7-*O*-glucoside were also purchased from Sigma Aldrich^®^–Merck^®^KGaA (Darmstadt, Germany). Malvidin-3-*O*-glucoside, delphinidin-3-*O*-galactoside, and cyanidin-3-*O*-glucoside were all purchased from Extrasynthese^®^ Genay CEDEX (Lyon, France).

### 3.2. Plant Material and Oilcake

*Pistacia lentiscus* L. (PL) fruits were harvested from ten wild adult plants growing in Maremma’s Park (Grosseto, Italy) at full maturation in December 2021. Fruits were collected in the field and transported to the laboratory in sealed zip-lock, tared plastic bags, and stored at −20 °C until the extraction process took place. After cleaning, ripe and unripe fruits were homogeneously mixed to obtain a pooled sample. The pooled sample was extracted using a cold-pressing procedure (KLARSTEIN, OL1-90300-duo, Berlin, Germany), following the conventional procedure for oil extraction of PL fruits. After, the oilcake was recovered and used as the starting material for the polyphenolic extraction (focused on anthocyanins and flavonols) using a UAE procedure. The oilcake was ground with liquid nitrogen and stored at −80 °C until the extraction.

### 3.3. General Conditions for the Oilcake Ultrasound-Assisted Extraction and Purification Steps

Ultrasound-assisted extraction (UAE) was performed with an ultrasonic apparatus (BioClass^®^ CP104) with the frequency set to 39 kHz and an input power of 100 W. A known amount of the oilcake samples was weighted using an analytical scale (Precisa^®^ 125A), and the extraction process was conducted following the conditions of the screening and optimization designs (see Section 2.2 in the Results and Discussion Section). Each extraction was divided into four UAE cycles to ensure an exhaustive extraction process.

After the extraction, the supernatant was defatted with n-hexane (1:3) to remove any lipophilic compounds and then dried at room temperature using a concentrator (Concentrator plus/Vacufuge^®^ plus 5305, Eppendorf AG, Hamburg, Germany). Subsequently, a two-stage purification process was employed to concentrate the oilcake extracts, yielding two enriched fractions: one primarily containing flavonols and the other rich in anthocyanins, which were the target compounds of interest in this study. To achieve this, the extracts were resuspended with acidic Milli-Q_H2O_ (pH = 2.5 adjusted with HCOOH) and partitioned with ethyl acetate (1:1), obtaining an ethyl acetate fraction (EF) and an aqueous fraction (AF). The latter was further purified by ion-exchange Solid Phase Extraction (SPE Bond Elut-SAX, 100 mg, 3 mL, Agilent Technologies, Santa Clara, CA, USA). The SPE were previously activated with methanol and washed with acidic water (2 mL, pH = 2.5 adjusted with HCOOH). Both the EF and the purified AF were dried using the concentrator and then resuspended in a 1:1 ratio of MeOH–Milli-Q_H2O_ (pH = 2.5 adjusted with formic acid HCOOH) to conduct the chromatographic analysis. The purification steps were conducted with the addition of a known amount of internal standards (oenin for the AF and caffeic acid for the EF) to verify and correct the possible variations in the anthocyanin and flavonol contents that might occur during the purification steps.

### 3.4. Quantification and Identification of Polyphenols: HPLC- DAD and LC-MS Analyses

#### 3.4.1. HPLC-DAD Analysis

High-performance liquid chromatography coupled with a diode array detector (HPLC-DAD) was employed to quantify the two classes of flavonoids of interest (flavonols and anthocyanins) in the oilcake extracts. The identification of the chemical compounds in the oilcake extract was based on the retention times (RT), UV–vis characteristics, comparisons with external standards, literature data, as well as the LC-MS analysis (see next section).

For the analysis, each sample was redissolved in a MeOH–Milli-Q_H20_ solution (1:1 *v*/*v*, pH = 2.55 for HCOOH) and injected into Perkin^®^ Elmer Flexar liquid chromatography equipment, equipped with a 200Q/410 quaternary pump and an LC 200 photodiode detector (DAD) (Perkin Elmer^®^, Branford, CT, USA). The stationary phase consisted of an Agilent Zorbax^®^ SB-C18 column (250 mm × 4.6 mm, particle size 5 μm) maintained at 30 °C during the analysis. Acidified water and a solution of MeOH–ACN (1:1 *v*/*v*), both with 1% HCOOH, were used as solvents A and B, respectively. Two different instrument gradient programs were applied. For the EF analysis, the following program was used: 5 min (5% B), 5–15 min (5–20% B), 15–40 min (20–50% B), 40–45 min (50% B), 45–55 min (50–80% B), and 55–60 min (80–5% B) for a total analysis time of 61 min using a flow rate of 0.4 mL min^−1^. For the AF analysis, the following gradient was used: 5 min (5% B), 5–25 min (5–20% B), 25–30 min (20% B), 30–45 min (20–25% B), 45–47 min (25–5% B), and 47–48 min (5% B) for a total analysis time of 49 min using a flow rate of 0.6 mL min^−1^.

The quantification of flavonoids was conducted using the HPLC-DAD calibration curves of the following standards: myricetin, rutin, quercetin-3,4-di-*O*-glucoside, quercetin-3-*O*-glucopyranoside, kaempferol-3-*O*-glucoside, kaempferol-7-*O*-glucoside, malvidin-3-*O*-glucoside, delphinidin-3-*O*-galactoside, and cyanidin-3-*O*-glucoside. When not commercially available, a representative compound was used as a standard for quantification. The calibration curve’s linearity was determined with the determination coefficient (R^2^), which was found to be greater than 0.999 for the regression equations of all of the calibration curves. Signal-to-noise ratios of 3 for the detection limit (LOD) and 10 for the quantification limit (LOQ) were established and are presented in micrograms per milliliter (μg/mL). The following values for the standards’ limits of detection (LOD) and quantification (LOQ) were obtained: LOD_rutin_ = 0.28 and LOQ_rutin_ = 0.6; LOD_myricitrin_ = 0.12 and LOQ_myricitrin_ = 0.38; and LOD_kaempferol-3-*O*-rutinoside_ = 0.21 and LOQ_kaempferol-3-*O*-rutinoside_ = 0.49).

The analyses were conducted in a wavelength range of 200 to 550 nm, and the EF and AF chromatograms were acquired at their maximum wavelength absorbance for each class: 350 and 520 nm for flavonols and anthocyanins, respectively. The quantification of the polyphenols was expressed mg g^−1^ of the dry oilcake extract. The total values were calculated from the sum of the individual compounds within each class: the Total Flavonol Content (TFC) was obtained from the EF, the Total Anthocyanin Content (TAC) was obtained from the AF, and their combined sum was reported as the Total Anthocyanin and Flavonol Content (TFAC).

#### 3.4.2. LC-MS Analysis

To identify the main compounds in both the AF and EF of the lentisk oilcake extracts, an LC-MS analysis was conducted. The analysis was carried out using high-performance liquid chromatography–ultraviolet detector–electrospray mass spectrometry (HPLC-UV-ESI-QqQ) using a Waters instrument (Waters Italy, Sesto S. Giovanni, Milan, Italy). The apparatus consisted of an Alliance 2695 HPLC with an autosampler, a column oven, and a Diode Array Detector 2996 coupled in series to a four-micro-triple quadrupole mass spectrometer equipped with a Z-spray ESI interface. The LC column was a Zorbax Eclipse Plus C18 column (100 × 2.1 mm, 1.8 µm; Agilent Technologies Italy, Cernusco s/N, Milan, Italy), and the separation conditions and the mobile phases were acidified water (solvent A) and a solution of MeOH–ACN (1:1 *v*/*v,* solvent B), both in 0.1% HCOOH. The elution program was the same as the one described in the previous section. The flow rate was set at 0.25 mL/min, and the column was kept at 40 °C. The injection volume was 20 µL, and no splitter was used between the UV and MS detectors. UV spectra were recorded in the 255–670 nm range at a 2.4 nm resolution and 0.5 spectrum/sec rate. The ESI interface and MS parameters were optimized using infusions of standard solutions. Positive and negative polarities were used, applying a source temperature of 120 °C and a desolvation gas temperature of 320 °C. High-purity nitrogen (N_2_) was used as the cone gas and desolvation gas at flow rates of 21 L/h and 345 L/h, respectively. In the positive ion mode, the spray capillary voltage was 3.2 kV and the extractor lens voltage was 3 V; 20 and 45 V were used for the cone voltage to reduce or promote source fragmentation. In the negative ion mode, the spray capillary voltage was 2.7 kV, and the extractor lens voltage was 3 V; two values, 18 and 45 V, were used for the cone voltage. The mass spectrometer was operated in full scan mode, and data were acquired in the *m*/*z* range of 230 to 1100 in the positive ion mode and 100 to 1250 in the negative ion mode, with a 1.2 s scan time and 0.1 s interscan delay. The phenolic compounds present in the samples were characterized according to their UV and mass spectra and retention times compared with commercial standards when available. To identify the compounds, a series of different tandem mass spectrometry scan modes were employed. Initially, a full MS scan was conducted to determine the molecular weight of the molecules. Following this, an MS/MS fragmentation analysis was carried out using a product ion scan, where the parent ions were selectively isolated in the first quadrupole and then fragmented in the collision cell. The identified compounds from the EF and AF are listed in Table 1 (Section 2.1.2, Results and Discussion Section).

### 3.5. Experimental Design for UAE Optimization Process: Screening and Optimization Steps

#### 3.5.1. Screening Design

A Fractional Factorial Design (FFD) was employed to systematically screen and select important variables for the extraction of polyphenols from the PL oilcake. Based on the literature and considering the green chemistry principles, four variables were chosen: time, temperature, solvent, and liquid-to-solid ratio. The 2^4−1^ FFD was chosen, using the four variables: time (T, in min, x_1_), temperature (t, in °C, x_2_), extraction solvent (S, Et-OH: Milli-Q_H20_ % *v*/*v*, x_3_), and liquid-to-solid ratio (LSR, in L g^−1^, x_4_). The experimental matrix was generated using Minitab^®^ 18 statistical software (Minitab Inc., State College, PA, USA). Each independent variable was tested at the minimum and maximum levels, coded as (−1) and (+1), respectively (see Table 2, Results and Discussion Section). The non-coded values for each independent variable were as follows: time (10 and 30 min), temperature (25 and 50 °C), extraction solvent (10 and 50% *v*/*v* EtOH), and liquid-to-solid ratio (0.01 and 0.02 L g^−1^). Nine experimental trials and one central point were randomly performed. The central point was conducted in triplicate, whereas the trials were performed in duplicate. The calculation of the coefficients associated with each variable (b1, b2, b3, and b4) allowed for the evaluation of the impact of each variable on the outputs, namely the Total Anthocyanin Content (TAC), Total Flavonol Content (TFC), and Total Flavonol and Anthocyanin Content (TFAC). The variables that exhibited significance in the regression analysis (*p* ≤ 0.05) were then chosen for the optimization design and construction of response surfaces (Response Surface Methodology—RSM).

#### 3.5.2. Optimization Design and Response Surface Methodology

The significant variables identified through the screening step (2^4−1^ FFD) were subjected to an optimization study using the Box–Behnken design (BBD) and statistically analyzed using a factorial ANOVA using Minitab^®^ 18 statistical software (Minitab Inc., State College, PA, USA). A three-level variable matrix was used to optimize the TAC, TFC, and TFAC in the PL oilcake extracts. The three independent variables were analyzed on three different levels, coded as (−1) for the lower level, (+1) for the higher level, and a middle level (0). The independent variables and their coded levels were as follows: time (x_1_; 5, 10, and 15 min), extraction solvent (x_2_; 30, 50, and 70% *v*/*v* EtOH), and liquid-to-solid ratio (x_4_; 0.008, 0.01, and 0.012 L g^−1^). Fifteen experimental trials were performed randomly with the central point tested in triplicate, following the conditions indicated by the BBD matrix. The BBD matrix with coded and uncoded variables, as well as outputs, is summarized in Table 2 (Results and Discussion Section). The output responses (TFC, TAC, and TFAC) were fitted into the quadratic polynomial equation (Equation (1)) to predict the optimal extraction conditions and construct response surfaces.

(1) $$\beta 0+\sum_{i=1}^{k}\beta_{i}X_{i}+\sum_{i=1}^{k}\beta_{ii}{X_{i}^{2}}_{ii}+\sum_{i=1}^{k}\sum_{j=i+1}^{k}\beta_{ij}Xij$$

where the equation terms are as follows: *Y*—response variables; *X_i_* and *X_j_*—independent variables; *β*0, *β_i_*, *β_ii_*, and *β_ij_*—regression coefficients of the model, namely the intercept, linear coefficient, quadratic coefficient, and interaction terms, respectively; and *k* is the number of variables (*k* = 3).

In order to assess the variability in the residuals in the proposed models, the lack of fit (LOF) test was conducted. Also, the estimated coefficients of multiple determinations for the quadratic models (R^2^) and their adjusted counterparts (R^2^adj), the coefficient of variation (CV), and Fisher’s test value (F-value) were calculated and evaluated; they were considered statistically significant when *p* ≤ 0.05. The data analysis was performed using Minitab^®^ 18 software, while the construction of the RSM resulting from the mathematical models was conducted using Statgraphics Centurion^®^ 19 (Statgraphics Technologies, Inc., The Plains, VA, USA), a comprehensive tool for data analysis. Following this, to substantiate the mathematical models’ predictive accuracy and reliability, a validation analysis was carried out through repetition of the optimal extraction conditions.

## 4. Conclusions

This study provided a comprehensive analysis of the ultrasound-assisted extraction (UAE) process for recovering anthocyanins and flavonols from a *Pistacia lentiscus* L. oilcake, using a combination of fractional factorial and Box–Behnken designs. The investigation revealed that the ethanol concentration and liquid-to-solid ratio (LSR) are the most influential parameters affecting the yield of these valuable polyphenolic compounds from PL oilcake extracts. Specifically, the optimal extraction conditions—15 min of extraction time, 70% *v*/*v* ethanol concentration, and an LSR of 0.012 L g^−1^—resulted in maximum yields of 19.78 mg g^−1^ extract for the total flavonol content and over 25.4 mg g^−1^ extract for the total anthocyanin and flavonol content (corresponding to 1.80 mg g^−1^ of dry oilcake for the TFC and 3.32 mg g^−1^ of dry oilcake for the TFAC, respectively)

The successful optimization of the parameters demonstrates the potential of UAE as a highly effective and efficient extraction technique, capable of maximizing both anthocyanin and flavonol recovery from this by-product. This study underscores the advantages of using advanced statistical methodologies to systematically explore and optimize extraction processes, thereby reducing the number of experimental trials required and ensuring resource-efficient research practices.

In addition, a key contribution of the study lies in the valorization of *Pistacia lentiscus* oilcakes, a traditionally underutilized plant by-product. Transforming this waste material into a valuable source of bioactive compounds highlights a sustainable approach to resource management. By repurposing oilcakes, which are typically discarded after oil extraction, our research showed that it might be possible to reduce environmental waste and add economic value to an important agricultural by-product. The successful extraction of these high-value compounds (anthocyanins and flavonols) from plant waste materials demonstrates the significant potential of this optimized methodology for various industries, including the food, cosmetics, and pharmaceutical industries.

Our study supports the broader goal of sustainable resource utilization by turning waste into wealth, showcasing how agricultural by-products can be repurposed effectively and sustainably. Additionally, UAE methods can be easily integrated into industrial settings due to their scalability and efficiency, further promoting the adoption of waste-derived materials in bio-based industries.

In conclusion, our study highlights the critical role of the ethanol concentration and quantity of plant material in UAE processes and validates the application of the Response Surface Methodology for optimizing complex extraction procedures. This scalability, combined with the high efficiency and reduced resource consumption of UAE processes, positions UAE as a promising technology. It can be applied to the large-scale production of bioactive compounds, reinforcing the role of modern extraction techniques in promoting sustainable and economically viable waste valorization strategies.

## Figures and Tables

**Table 1:** Anthocyanins and flavonols in the PL oilcake extract fractions AF and EF, respectively. The peak numbers correspond to the chromatographic profile illustrated in Figure 2a for the AF and Figure 2b for the EF.

Peak	Compound Assignment	RT (min)	UV-vis λ_max_ (nm)	Molecular Ion (*m/z*)	Fragment Ion (*m/z*)	Ionization (+/−)	Collision Energy (V)
	**Anthocyanins**						
1	Delphinidin di-hexoside 1	21.1	283, 430 sh, 519	627	465, 303	+	35
2	Delphinidin di-hexoside 2	21.6	283, 430 sh, 519	627	465, 303	+	35
3	(*) Delphinidin-3-*O*-galactoside	25.1	280, 435 sh, 526	465	303	+	30
4	(*) Delphinidin-3-*O*-glucoside	26.2	280, 432 sh, 525	465	303	+	35
5	Cyanidin-3-*O*-galactoside	27.9	281, 434 sh, 518	449	287	+	25
6	Delphinidin pentoside	29.3	281, 355, 520	435	303	+	35
7	(*) Cyanidin-3-*O*-glucoside	31.9	281, 353, 525	449	287	+	25
8	Cyanidin pentoside	33.9	282, 354, 527	419	287	+	25
	**Flavonols**						
1	Myricetin rutinoside	31.04	269, 298 sh, 358	625	479, 317	-	
2	Myricetin glucuronide	31.2	269, 300 sh, 360	493	317	-	25
3	Myricetin-hexoside isomer 1	33.3	269, 300 sh, 361	479	317	-	25
4	Myricetin-hexoside isomer 2	33.5	269, 300 sh, 362	479	317	-	25
5	(*) Quercetin-3-*O*-glucoside isomer 1	34.2	278, 309 sh, 350	463	301	-	55
6	Quercetin-3-*O*-glucoside isomer 2	34.7	278, 308 sh, 350	463	301	-	55
7	Myricetin pentoside	35.4	278, 300 sh, 364	449	317	-	20
8	Quercitrin	35.5	276, 302 sh, 368	447	301	-	30
9	(*) Rutin	36.2	270, 300 sh, 355	609,	477, 301	-	50
10	Myricitrin	37.7	277, 303 sh, 360	463	317	-	25
11	Quercetin arabinoside	38.9	275, 300 sh, 364	433	867, 301	-	50
12	Kaempferol derivative	39.5	275, 300 sh, 400	/	285	-	18
13	Myricetin derivative	40.2	272, 300 sh, 355	469	317	-	20
14	Kaempferol derivative	41.0	264, 300 sh, 370	/	285	-	10
15	Flavonoid derivative	42.6	273, 404	/	269	-	15
16	Kaempferol derivative	45.4	272, 298 sh, 405	/	285	-	10
17	Kaempferol derivative	47.4	256, 300 sh, 372	/	285	-	10
18	Quercetin aglycone	48	269,280 sh, 347	/	301	-	55
19	Kaempferol derivative	50.7	270, 282 sh, 350	/	285	-	10
20	Kaempferol derivative	54	268, 280 sh, 352	/	285	-	10

(*) identified using external standard; sh: shoulder.

**Table 2:** Fractional Factorial Design matrix (2^4−1^), variables (x_1_ to x_4_), and corresponding calculated coefficients (b_1_ to b_4_).

Independent Variables (x)								
Run	x_1_ Temperature (°C)		x_2_ Time (min)		x_3_ Solvent (% EtOH)		x_4_ Liquid/Solid Ratio (L g^−1^)	
1	25	−	10	−	10	−	0.01	−
2	50	+	10	−	10	−	0.02	+
3	25	−	30	+	10	−	0.02	+
4	50	+	30	+	10	−	0.01	−
5	25	−	10	−	50	+	0.02	+
6	50	+	10	−	50	+	0.01	−
7	25	−	30	+	50	+	0.01	−
8	50	+	30	+	50	+	0.02	+
9	37	0	20	0	30	0	0.015	0
Output (Y)				Coefficients				
TFC	b_1_	−0.960	b_2_	−4.500 *	b_3_	−1.920	b_4_	−5.810 **
TAC	b_1_	0.226	b_2_	0.128	b_3_	0.857 ***	b_4_	−0.031
TFAC	b_1_	−0.730	b_2_	−4.730 *	b_3_	−1.060	b_4_	−5.840 *

Each coefficient (b1–b4) is associated with a corresponding variable (x_1_–x_4_). Statistically significant results are reported as follows: * for *p* ≤ 0.05, ** for *p* ≤ 0.01, and *** for *p* ≤ 0.001. Total Flavonol Content (TFC), Total Anthocyanin Content (TAC), and Total Flavonol and Anthocyanin Content (TFAC).

**Table 3:** Box–Behnken design matrix with coded and uncoded values of the variables (x_1_ to x_3_), and their corresponding calculated coefficients (b_1_ to b_4_) and outputs. Results are expressed as mg g^−1^ of PL oilcake extract.

Run	Independent Variables (x)						Outputs (Y)		
	x_1_ Time (min)		x_2_ Solvent (% EtOH)		x_3_ Liquid/Solid Ratio (L g^−1^)		TFC	TAC	TFAC
1	5	−	30	−	0.01	0	9.7	4.4	14.2
2	15	+	30	−	0.01	0	9.8	4.1	14.0
3	5	−	70	+	0.01	0	19.0	5.2	24.2
4	15	+	70	+	0.01	0	19.8	5.2	25.0
5	5	−	50	0	0.008	−	15.7	5.0	20.7
6	15	+	50	0	0.008	−	15.2	4.9	20.1
7	5	−	50	0	0.012	+	14.0	4.6	18.6
8	15	+	50	0	0.012	+	16.1	5.2	21.3
9	10	0	30	−	0.008	−	9.0	4.2	13.1
10	10	0	70	+	0.008	−	17.1	5.0	22.1
11	10	0	30	−	0.012	+	9.6	3.6	13.3
12	10	0	70	+	0.012	+	18.1	4.5	22.5
13	10	0	50	0	0.01	0	14.2 ± 0.5	4.9 ± 0.1	19.1 ± 0.3
Output (y)	Coefficients								
TFC	b1	0.301	b2	4.478 *	b3	0.102			
TAC	b1	0.037	b2	0.442 *	b3	−0.141			
TFAC	b1	0.337	b2	4.921 *	b3	−0.039			

Total Flavonol Content (TFC), Total Anthocyanin Content (TAC), and Total Flavonol and Anthocyanin Content (TFAC). Central point outputs are expressed as mean ± standard deviation (n = 3). Statistically significant results are indicated by * for *p* ≤ 0.05.

**Table 4:** Statistical and fit analysis of the models obtained for the different responses (TFC, TAC, and TFAC).

Output	Model Analysis			Lack of Fit (LOF)		
	R^2^	R^2^ Adj	F-Value of Model	*p*-Value of Model	F-Value of LOF	*p*-Value of LOF
TFC	0.99	0.96	43.36	0.001 *	2.55	0.294
TAC	0.92	0.80	6.61	0.026 *	3.84	0.214
TFAC	0.99	0.98	64.77	0.001 *	4.37	0.192

TFC (Total Flavonol Content), TAC (Total Anthocyanin Content), and TFAC (Total Flavonol Anthocyanin Content). ns: not significant. Statistically significant results are indicated by *, for *p* ≤ 0.05.

## Data Availability

The data presented in this study are available on request from the corresponding author.

## Supplementary Materials

The following supporting information can be downloaded at https://www.mdpi.com/article/10.3390/molecules30020237/s1. Table S1: Total individual anthocyanins from AF (mg g^−1^ of lentisk oilcake dry extract) corresponding to peaks 1–8 in Figure 1a and Table 1 (results and discussion section) obtained in Box Behnken Design step; Table S2: Total individual flavonols from EF (mg g^−1^ of lentisk oilcake dry extract) corresponding to peaks 1–20 in Figure 1b and Table 1 (Results and Discussion Section) obtained in Box Behnken Design step; Table S3: Columns represent the regression coefficients for the intercept, linear, quadratic, and interaction terms in the respective models on Box Behnken design. TFC (Total Flavonoid Content), TAC (Total Anthocyanins Content), and TFAC (Total Flavonoid and Anthocyanins Content); Table S4: Dry extract yield % obtained in Box-Behnken Design starting from dry oilcake and the corresponding quantification of TFC, TAC, and TFAC reported as mg g^−1^ of oilcakes.
