# Correction: Muti et al. From Waste to Value: Optimization of Ultrasound-Assisted Extraction of Anthocyanins and Flavonols from *Pistacia lentiscus* L. Oilcakes. *Molecules* 2025, *30*, 237

**DOI:** 10.3390/molecules30132666

**Published:** 2025-06-20

**Authors:** Lucrezia Muti, Luana Beatriz dos Santos Nascimento, Giulia Goracci, Cassandra Detti, Cecilia Brunetti, Anna Rita Bilia, Francesco Ferrini, Antonella Gori

**Affiliations:** 1 Department of Agriculture, Food, Environment and Forestry (DAGRI), University of Florence, Sesto Fiorentino, 50019 Florence, Italy; lucrezia.muti@unifi.it (L.M.); cassandra.detti@unifi.it (C.D.); cecilia.brunetti@ipsp.cnr.it (C.B.); francesco.ferrini@unifi.it (F.F.); antonella.gori@unifi.it (A.G.); 2 Department of Chemistry, University of Florence, Via U. Schiff 6, Sesto Fiorentino, 50019 Florence, Italy; giulia.goracci@unifi.it (G.G.); ar.bilia@unifi.it (A.R.B.); 3 Institute for Sustainable Plant Protection (IPSP), National Research Council of Italy, Sesto Fiorentino, 50019 Florence, Italy

## Error in Table

In the original publication [1], there was a mistake in Table 1 as published. Minor mistakes in the identification of some peaks were made. The corrected Table 1 appears below.

## Text Correction

There were minor errors in the original publication [1], regarding the putative identification of kaempferol derivatives.

A correction has been made to 2. Results and Discussion Section, Section 2.1.2. Identified Flavonols from Ethylacetate Fraction and Section 2.2.2. Optimization Design and Response Surface Methodology.

The 4th sentence in Section 2.1.2, Paragraph 8 should be changed as: “Based on the fragmentation pattern, peak 12 was tentatively identified as kaempferol derivative, showing the fragment ion [M-H]^−^ at *m/z* 285.” 

The Section 2.1.2 Paragraphs 9 and 10 should be changed as: “The identity of peak 13 instead was tentatively assigned with myricetin-derivative, presenting a precursor ion [M-H]^−^ at *m*/*z* 469 and a fragment ion [M-H]^−^ at *m*/*z* 317, with a neutral loss of *m*/*z* 152. Peaks 14 and 16 were putatively identified as kaempferol derivatives, sharing the fragment ion [M-H]^−^ at *m*/*z* 285 corresponding to kaempferol aglycone, while peak 15 has been putatively identified as a flavonoid derivative with the main ion [M-H]^−^ at *m*/*z* 269. Peak 18 was identified as quercetin aglycone, with a [M-H]^−^ signal at *m*/*z* 301. Lastly, peaks 17, 19, and 20 were assigned as kaempferol derivatives, as they showed the kaempferol aglycone [M-H]^−^ at *m*/*z* 285 and similar UV–Vis spectra.”

The 2nd sentence in Section 2.2.2, Paragraph 2 should be changed as: “Among the tested conditions, run 4 (15 347 min, 70% EtOH, and 0.01 g L^−1^) yielded the highest TFC and TAC values, alongside the highest TFAC, with cyanidin-3-*O*-galactoside and kaempferol derivative being the most abundant compounds in the AF and EF, respectively.” 

The Supplementary Materials has been corrected accordingly.

The authors state that the scientific conclusions are unaffected. This correction was approved by the Academic Editor. The original publication has also been updated.

## Figures and Tables

**Table 1:** Anthocyanins and flavonols in the PL oilcake extract fractions AF and EF, respectively. The peak numbers correspond to the chromatographic profile illustrated in Figure 2a for the AF and Figure 2b for the EF.

Peak	Compound Assignment	RT (min)	UV-vis λ_max_ (nm)	Molecular Ion (*m/z*)	Fragment Ion (*m/z*)	Ionization (+/−)	Collision Energy (V)
	**Anthocyanins**						
1	Delphinidin di-hexoside 1	21.1	283, 430 sh, 519	627	465, 303	+	35
2	Delphinidin di-hexoside 2	21.6	283, 430 sh, 519	627	465, 303	+	35
3	(*) Delphinidin-3-*O*-galactoside	25.1	280, 435 sh, 526	465	303	+	30
4	(*) Delphinidin-3-*O*-glucoside	26.2	280, 432 sh, 525	465	303	+	35
5	Cyanidin-3-*O*-galactoside	27.9	281, 434 sh, 518	449	287	+	25
6	Delphinidin pentoside	29.3	281, 355, 520	435	303	+	35
7	(*) Cyanidin-3-*O*-glucoside	31.9	281, 353, 525	449	287	+	25
8	Cyanidin pentoside	33.9	282, 354, 527	419	287	+	25
	**Flavonols**						
1	Myricetin rutinoside	31.04	269, 298 sh, 358	625	479, 317	-	
2	Myricetin glucuronide	31.2	269, 300 sh, 360	493	317	-	25
3	Myricetin-hexoside isomer 1	33.3	269, 300 sh, 361	479	317	-	25
4	Myricetin-hexoside isomer 2	33.5	269, 300 sh, 362	479	317	-	25
5	(*) Quercetin-3-*O*-glucoside isomer 1	34.2	278, 309 sh, 350	463	301	-	55
6	Quercetin-3-*O*-glucoside isomer 2	34.7	278, 308 sh, 350	463	301	-	55
7	Myricetin pentoside	35.4	278, 300 sh, 364	449	317	-	20
8	Quercitrin	35.5	276, 302 sh, 368	447	301	-	30
9	(*) Rutin	36.2	270, 300 sh, 355	609,	477, 301	-	50
10	Myricitrin	37.7	277, 303 sh, 360	463	317	-	25
11	Quercetin arabinoside	38.9	275, 300 sh, 364	433	867, 301	-	50
12	Kaempferol derivative	39.5	275, 300 sh, 400	/	285	-	18
13	Myricetin derivative	40.2	272, 300 sh, 355	469	317	-	20
14	Kaempferol derivative	41.0	264, 300 sh, 370	/	285	-	10
15	Flavonoid derivative	42.6	273, 404	/	269	-	15
16	Kaempferol derivative	45.4	272, 298 sh, 405	/	285	-	10
17	Kaempferol derivative	47.4	256, 300 sh, 372	/	285	-	10
18	Quercetin aglycone	48	269,280 sh, 347	/	301	-	55
19	Kaempferol derivative	50.7	270, 282 sh, 350	/	285	-	10
20	Kaempferol derivative	54	268, 280 sh, 352	/	285	-	10

(*) identified using external standard; sh: shoulder.
