# Supplementary material for: From Waste to Value: Optimization of Ultrasound-Assisted Extraction of Anthocyanins and Flavonols from Pistacia lentiscus L. Oilcakes
Source: Molecules. 2025 Jan 9;30(2):237. doi: 10.3390/molecules30020237 (PMC11767306; doi:10.3390/molecules30020237)
Supplement: Supplementary file 1 [file molecules-30-00237-s001.zip › SUPPLEMENTARY MATERIALS_final version_corrected.pdf]

SUPPLEMENTARY MATERIALS

From Waste to Value: Optimization of Ultrasound-Assisted Extraction of Anthocyanins and Flavonols from *Pistacia lentiscus* L. Oilcakes

Lucrezia Muti [1], Luana Beatriz dos Santos Nascimento [1,*,†], Giulia Goracci [2], Cassandra Detti [1], Cecilia Brunetti [1,3], Anna Rita Bilia [2], Francesco Ferrini [1,3] and Antonella Gori [1,3]

[1] Department of Agriculture, Food, Environment and Forestry (DAGRI), University of Florence, Sesto Fiorentino, 50019 Florence, Italy; lucrezia.muti@unifi.it (L.M.); cassandra.detti@unifi.it (C.D.); francesco.ferrini@unifi.it (F.F.); antonella.gori@unifi.it (A.G.)
[2] Department of Chemistry, University of Florence, Via U. Schiff 6, Sesto Fiorentino, 50019 Florence, Italy; giulia.goracci@unifi.it (G.G.); ar.bilia@unifi.it (A.R.B.)
[3] Institute for Sustainable Plant Protection (IPSP), National Research Council of Italy, Sesto Fiorentino, 50019 Florence, Italy; cecilia.brunetti@ipsp.cnr.it (C.B.)
[*] Correspondence: luanabeatriz.dossantosnascimento@unifi.it or luananascimento@ufrj.br
[†] Current address: Centro de Ciências da Saúde, Federal University of Rio de Janeiro, Avenida Carlos Chagas Filho, 21941-599, Rio de Janeiro, Brazil.

Table S1: Total individual anthocyanins from AF (mg g-1of lentisk oilcake dry extract) corresponding to peaks 1-8 in Figure 1a and Table 1 (results and discussion section) obtained in Box Behnken Design step

Runs	x1	x2	x3	Peak 1-2 (Del-dHex1/2)	Peak 3 (Del3Ogal)	Peak 4 (Del3Oglu)	Peak 5 (Cya3Oglu	Peak 6 (Del-pent)	Peak 7 (Cya3Oglu)	Peak 8 (Cya-pent)	TAC
**1**	**5**	**30**	**0.01**	0.35 ± 0.02	0.48 ± 0.003	0.35 ± 0.003	2.14 ± 0.06	0.29 ± 0.0004	0.64 ± 0.02	0.17 ± 0.02	4.43 ± 0.04
**2**	**15**	**30**	**0.01**	0.34 ± 0.05	0.44 ± 0.6	0.34 ± 0.05	1.97 ± 0.23	0.27 ± 0.04	0.65 ± 0.07	0.12 ± 0.02	4.14 ± 0.49
**3**	**5**	**70**	**0.01**	0.34 ± 0.02	0.60 ± 0.02	0.40 ± 0.02	2.61 ± 0.05	0.31 ± 0.01	0.75 ± 0.01	0.18 ± 0.02	5.19 ± 0.10
**4**	**15**	**70**	**0.01**	0.34 ± 0.008	0.59 ± 0.03	0.40 ± 0.02	2.64 ± 0.12	0.31 ± 0.01	0.78 ± 0.01	0.17 ± 0.01	5.24 ± 0.22
**5**	**5**	**50**	**0.008**	0.33 ± 0.02	0.56 ± 0.02	0.39 ± 0.02	2.48 ± 0.08	0.30 ± 0.01	0.75 ± 0.03	0.16 ± 0.02	4.97 ± 0.15
**6**	**15**	**50**	**0.008**	0.36 ± 0.007	0.54 ± 0.02	0.39 ± 0.03	2.40 ± 0.01	0.31 ± 0.01	0.75 ± 0.002	0.16 ± 0.03	4.91 ± 0.02
**7**	**5**	**50**	**0.012**	0.34 ± 0.06	0.52 ± 0.02	0.35 ± 0.01	2.32 ± 0.04	0.28 ± 0.002	0.67 ± 0.004	0.15 ± 0.03	4.61 ± 0.10
**8**	**15**	**50**	**0.012**	0.39 ± 0.03	0.59 ± 0.02	0.41 ± 0.002	2.58 ± 0.05	0.32 ± 0.01	0.75 ± 0.02	0.16 ± 0.02	5.21 ± 0.05
**9**	**10**	**30**	**0.008**	0.36 ± 0.02	0.45 ± 0.04	0.34 ± 0.03	1.96 ± 0.19	0.27 ± 0.03	0.61 ± 0.07	0.17 ± 0.03	4.15 ± 0.41
**10**	**10**	**70**	**0.008**	0.32 ± 0.04	0.58 ± 0.06	0.40 ± 0.02	2.56 ± 0.29	0.32 ± 0.01	0.65 ± 0.05	0.15 ± 0.01	4.99 ± 0.46

**11**	**10**	**30**	**0.012**	0.27 ± 0.12	0.43 ± 0.06	0.34 ± 0.01	1.82 ± 0.38	0.27 ± 0.001	0.44 ± 0.18	nd	3.62 ± 0.84
**12**	**10**	**70**	**0.012**	0.26 ± 0.17	0.55 ± 0.14	0.42 ± 0.02	2.31 ± 0.79	0.33 ± 0.01	0.60 ± 0.31	nd	4.46 ± 1.43
**13**	**10**	**50**	**0.01**	0.26 ± 0.13	0.53 ± 0.05	0.42 ± 0.02	2.06 ± 0.43	0.33 ± 0.003	0.50 ± 0.24	nd	4.10 ± 0.81

Del-dHex1/2 – delphinidin di-hexoside isomer 1 and 2; Del3Ogal – delphinidin-3 O-galactoside; Del3Ogluc – delphinidin-3-O-glucoside; Cya3Ogluc – cyanidin-3-O-glucoside; Del-pent – delphinidin pentoside; TAC–Total Anthocyanin Content

Table S2: Total individual flavonols from EF (mg $g^{-1}$of lentisk oilcake dry extract) corresponding to peaks 1-20 in Figure 1b and Table 1 (Results and discussion section) obtained in Box Behnken Design step.

Runs	x1	x2	x3	Peak 1-2 (MYR-Rut; MYR-Glc)	Peak 3 (MYR-Hex1)	Peak 4 (MYR-hex2)	Peak 5 (QUE-3OGluc1)	Peak 6 (QUE-3OGluc2)	Peak 7-8 (MYR-P/QUER)	Peak 9 (RUT)	Peak 10 (MYRC)
**1**	**5**	**30**	**0.01**	0.87± 0.02	0.23± 0.002	0.1± 0.001	0.61 ± 0.03	0.07 ± 0.006	1.84 ± 0.003	0.52 ± 0.04	1.079 ± 0.02
**2**	**15**	**30**	**0.01**	0.087± 0.03	0.22 ± 0.004	0.09 ± 0.004	0.6 ± 0.03	0.08 ± 0.004	1.78 ± 0.0009	0.42 ± 0.02	1.02 ± 0.04
**3**	**5**	**70**	**0.01**	1.13 ± 0.08	0.26 ± 0.005	0.16 ± 0.02	0.78± 0.006	0.09 ± 0.0005	2.47 ± 0.09	0.77 ± 0.03	1.60 ± 0.01
**4**	**15**	**70**	**0.01**	0.93 ± 0.3	0.19 ± 0.073	0.17 0.05	0.69 ± 0.19	0.08 ± 0.02	2.08 ± 0.65	0.59 ± 0.21	1.31 ± 0.03
**5**	**5**	**50**	**0.008**	1 ± 0.03	0.23 ± 0.01	0.14 ± 0.007	0.73 ± 0.01	0.08 ± 0.009	2.22 ± 0.07	0.68 ± 0.01	1.40 ± 0.04
**6**	**15**	**50**	**0.008**	1.02 ± 0.003	0.24 ± 0.01	0.13 ± 0.03	0.71 ± 0.02	0.12 ± 0.01	2.25 ± 0.01	0.72 ± 0.09	1.39 ± 0.01
**7**	**5**	**50**	**0.012**	0.94 ±0.03	0.22 ±0.001	0.13 ± 0.02	0.67 ± 0.01	0.10 ± 0.007	2.08 ± 0.06	0.65 ± 0.05	1.32 ± 0.03
**8**	**15**	**50**	**0.012**	1.04 ± 0.04	0.23 ± 0.01	0.18 ± 0.04	0.72 ± 0.03	0.10 ± 0.002	2.26 ± 0.007	0.65 ± 0.07	1.43± 0.03
**9**	**10**	**30**	**0.008**	0.089 ± 0.008	0.023 ± 0.005	0.08 ± 0.001	0.60 ± 0.02	0.09 ± 0.005	1.78 ± 0.06	0.38 ± 0.03	1.00 ± 0.02
**10**	**10**	**70**	**0.008**	1.15 ± 0.02	0.26 ± 0.01	0.21 ± 0.03	0.86 ± 0.05	0.10 ± 0.02	2.54 ± 0.04	0.86 ± 0.1	1.617 ± 0.02
**11**	**10**	**30**	**0.012**	0.84 ± 0.03	0.20 ± 0.005	0.1 ± 0.01	0.60 ± 0.03	0.09 ± 0.01	1.73 ± 0.02	0.44 ± 0.07	1.017 ± 0.03
**12**	**10**	**70**	**0.012**	1.19 ± 0.05	0.25 ± 0.008	0.19 ± 0.03	0.90 ± 0.006	0.1 ± 0.004	2.59 ± 0.04	0.89 ± 0.01	1.668 ± 0.04
**13**	**10**	**50**	**0.01**	0.96 ± 0.03	0.22 ± 0.01	0.17 ± 0.01	0.73 ± 0.03	0.1 ± 0.006	2.12 ± 0.04	0.67 ± 0.06	1.337 ±0.02

Table S2. cont.: Total individual flavonols from EF (mg g^{-1}of lentisk oilcake dry extract) corresponding to peaks 1-20 in Figure 1b and Table 1 (Results and discussion section) obtained in Box Behnken Design step.

Runs	x1	x2	x3	Peak 11 (QUE-Ar)	Peak 12 (KAE-dv)	Peak 13 (MYR-dv)	Peak 14 (KAE-dv)	Peak 15 (Flav)	Peak 16 (KAE-dv)	Peak 17 (KAE-dv)	Peak 18 (QUE-a)	Peak 19 (KAE-dv)	Peak 20 (KAE-dv)	TFC
**1**	**5**	**30**	**0.01**	0.78 ± 0.07	1.08 ± 0.11	0.18 ± 0.005	0.29 ± 0.002	0.11 ± 0.01	0.29 ± 0.02	0.56 ± 0.01	0.56 ± 0.02	0.2 ± 0.02	0.12 ± 0.003	9.72 ± 0.17
**2**	**15**	**30**	**0.01**	0.82 ± 0.11	1.19 ± 0.17	0.17 ± 0.01	0.32 ± 0.01	0.13 ± 0.02	0.32 ± 0.04	0.60 ± 0.03	0.62 ± 0.13	0.21 ± 0.02	0.13 ± 0.005	9.83 ± 0.24
**3**	**5**	**70**	**0.01**	0.98 ± 0.011	4.01 ± 0.4	0.3 ± 0.06	0.29 ± 0.04	1.17 ± 0.18	0.1 ± 0.24	0.27 ± 0.003	2.48 ± 0.32	0.26 ± 0.001	0.31 ± 0.03	19.04 ± 1.39
**4**	**15**	**70**	**0.01**	0.76 ± 0.12	3.42 ± 1.40	0.26 ± 0.07	0.24 ± 0.09	1.01 ± 0.56	0.77 ± 0.25	0.22 ± 0.05	2.00 ± 0.88	0.24 ± 0.1	0.26 ± 0.09	15.78 ± 3.81
**5**	**5**	**50**	**0.008**	0.8 ± 0.005	3.11 ± 0.16	0.29 ± 0.01	0.24 ± 0.03	0.89 ± 0.02	0.77 ± 0.12	0.28 ± 0.02	1.90 ± 0.18	0.23 ± 0.003	0.24 ± 0.01	13.70 ± 0.19
**6**	**15**	**50**	**0.008**	0.83 ± 0.02	2.97 ± 0.64	0.24 ± 0.03	0.23 ± 0.003	0.70 ± 0.21	0.71 ± 0.0006	0.34 ± 0.06	1.71 ± 0.33	0.22 ± 0.01	0.22 ± 0.03	15.20 ± 1.16
**7**	**5**	**50**	**0.012**	0.79 ± 0.03	2.63 ± 0.28	0.22 ± 0.05	0.20 ± 0.01	0.58 ± 0.08	0.70 ± 0.2	0.30 ± 0.05	1.60 ± 0.14	0.22 ± 0.001	0.21 ± 0.02	14.01 ± 0.80
**8**	**15**	**50**	**0.012**	0.90 ± 0.11	3.17 ± 0.54	0.24 ± 0.06	0.25 ± 0.02	0.69 ± 0.16	0.85 ± 0.14	0.39 ± 0.12	1.90 ± 0.25	0.25 ± 0.003	0.26 ± 0.02	16.06 ± 0.99
**9**	**10**	**30**	**0.008**	0.73 ± 0.1	0.88 ± 0.11	0.18 ± 0.001	0.26 ± 0.004	0.08 ± 0.03	0.24 ± 0.02	0.52 ± 0.03	0.54 ± 0.05	0.19 ± 0.003	0.12 ± 0.008	8.96 ± 0.08
**10**	**10**	**70**	**0.008**	1.02 ± 0.13	3.07 ± 0.55	0.29 ± 0.03	0.22 ± 0.003	0.65 ± 0.15	0.90 ± 0.006	0.32 ± 0.08	1.94 ± 0.34	0.25 ± 0.08	0.26 ± 0.04	17.27 ± 0.99
**11**	**10**	**30**	**0.012**	0.71 ± 0.05	1.27 ± 0.12	0.17 ± 0.01	0.27 ± 0.03	0.15 ± 0.01	0.33 ± 0.03	0.53 ± 0.009	0.65 ± 0.03	0.20 ± 0.003	0.13 ± 0.0008	9.64 ± 0.21
**12**	**10**	**70**	**0.012**	0.97 ± 0.06	3.60 ± 0.14	0.27 ± 0.05	0.23 ± 0.01	0.83 0.16	0.87 ± 0.03	0.30 ± 0.009	2.11 ± 0.15	0.24 ± 0.004	0.26 ± 0.03	18.06 ± 0.68
**13**	**10**	**50**	**0.01**	0.83 ± 0.1	2.52 ± 0.28	0.23 ± 0.03	0.24 ± 0.03	0.57 ± 0.11	0.73 ± 0.16	0.39 ± 0.09	1.47 ± 0.13	0.24 ± 0.03	0.20 ± 0.04	14.22 ± 0.60

MYR-Rut – myricetin rutinoside; MYR-Glc – myricetin glucuronide; MYR-Hex1 –myricetin hexoside isomer 1 ; MYR-hex2 –myricetin hexoside isomer 2; QUE-3OGluc1 – quercetin-3-*O*-glucoside isomer 1; QUE-3OGluc2 – quercetin-3-*O*-glucoside isomer 2; MYR-P – myricetin pentoside; QUER – quercitrin; RUT– rutin; MYRC – myricitrin QUE-Ar – quercetin arabinoside; ; MYR-dv – myricetin derivative; KAE-dv – kaempferol derivative; QUE-a – quercetin aglycone; TFC Total Flavonids Content

Table S3. Columns represent the regression coefficients for the intercept, linear, quadratic, and interaction terms in the respective models on Box Behnken design. TFC (Total Flavonoid Content), TAC (Total Anthocyanins Content), and TFAC (Total Flavonoid and Anthocyanins Content).

Term for each response	Coefficients	Standard error	p-value
**TFC** = $14.224 + 0.301\ x1 + 4.478\ x2 + 0.102\ x3 + 1.084\ x1^2 – 0.715\ x2^2 – 0.066\ x3^2 + 0.158\ x1x2 + 0.634\ x1x3 + 0.068\ x2x3$			
b0	14.224	0.381	0.000*
b1	0.301	0.233	0.254
b2	4.478	0.233	0.000*
b3	0.102	0.233	0.680
b11	1.084	0.343	0.025*
b22	-0.715	0.343	0.092
b33	-0.066	0.343	0.856
b12	0.158	0.330	0.652
b13	0.634	0.330	0.113
b23	0.068	0.330	0.844
**TAC** = $4.882 + 0.0365\ x1 + 0.4428\ x2 – 0.1409\ x3 + 0.244\ x1^2 - 0.375\ x2^2 – 0.202\ x3^2 + 0.086\ x1x2 + 0.165\ x1x3 + 0.002\ x2x3$			
b0	4.882	0.125	0.000*
b1	0.0365	0.0768	0.655
b2	0.4428	0.0768	0.002*
b3	-0.1409	0.0768	0.126
b11	0.244	0.113	0.083
b22	-0.375	0.113	0.021*
b33	-0.202	0.113	0.134
b12	0.086	0.109	0.462
b13	0.165	0.109	0.190
b23	0.002	0.109	0.986
**TFAC** = $19.106 + 0.337\ x1 + 4.921\ x2 – 0.039\ x3 + 1.328\ x1^2 - 1.090\ x2^2 – 0.267\ x3^2 + 0.244\ x1x2 + 0.799\ x1x3 + 0.07\ x2x3$			
b0	19.106	0.346	0.000*
b1	0.337	0.212	0.173
b2	4.921	0.212	0.000*
b3	-0.039	0.212	0.861
b11	1.328	0.312	0.008*
b22	-1.090	0.312	0.017*
b33	-0.267	0.312	0.430
b12	0.244	0.300	0.452

b13	0.799	0.300	0.045*
b23	0.070	0.300	0.824

Statistically significant results are indicated as (*) for $p \leq 0.05$

Table S4: Dry extract yield % obtained in Box-Behnken Design starting from dry oilcake and the corresponding quantification of TFC, TAC, and TFAC reported as mg g^{-1} of oilcakes

Run	x1	x2	x3	Yield %	TAC	TFC	TAFC
1	5	30	0.01	9.81	0.43	0.95	1.39
2	15	30	0.01	9.97	0.41	0.98	1.40
3	5	70	0.01	9.15	0.48	1.74	2.21
4	15	70	0.01	9.12	0.47	1.81	2.28
5	5	50	0.008	9.94	0.50	1.56	2.06
6	15	50	0.008	9.57	0.47	1.45	1.92
7	5	50	0.012	10.89	0.50	1.52	2.03
8	15	50	0.012	9.89	0.51	1.59	2.11
9	10	30	0.008	9.33	0.39	0.84	1.22
10	10	70	0.008	8.67	0.43	1.48	1.92
11	10	30	0.012	11.36	0.41	1.09	1.51
12	10	70	0.012	9.28	0.42	1.68	2.09
13	10	50	0.01	10.23	0.50	1.45	1.95
